# Supplementary material for: Magneto-active substrates for local mechanical stimulation of living cells
Source: Sci Rep. 2018 Jan 23;8:1464. doi: 10.1038/s41598-018-19804-1 (PMC5780514; doi:10.1038/s41598-018-19804-1)
Supplement: Supplementary file 1 — Supplementary information [file 41598_2018_19804_MOESM1_ESM.pdf]

Magneto-active substrates for local mechanical stimulation of living cells

Cécile M. Bidan, Mario Fratzl, Alexis Coullomb, Philippe Moreau, Alain H. Lombard, Irène Wang, Martial Balland, Thomas Boudou, Nora M. Dempsey, Thibaut Devillers* and Aurélie Dupont*

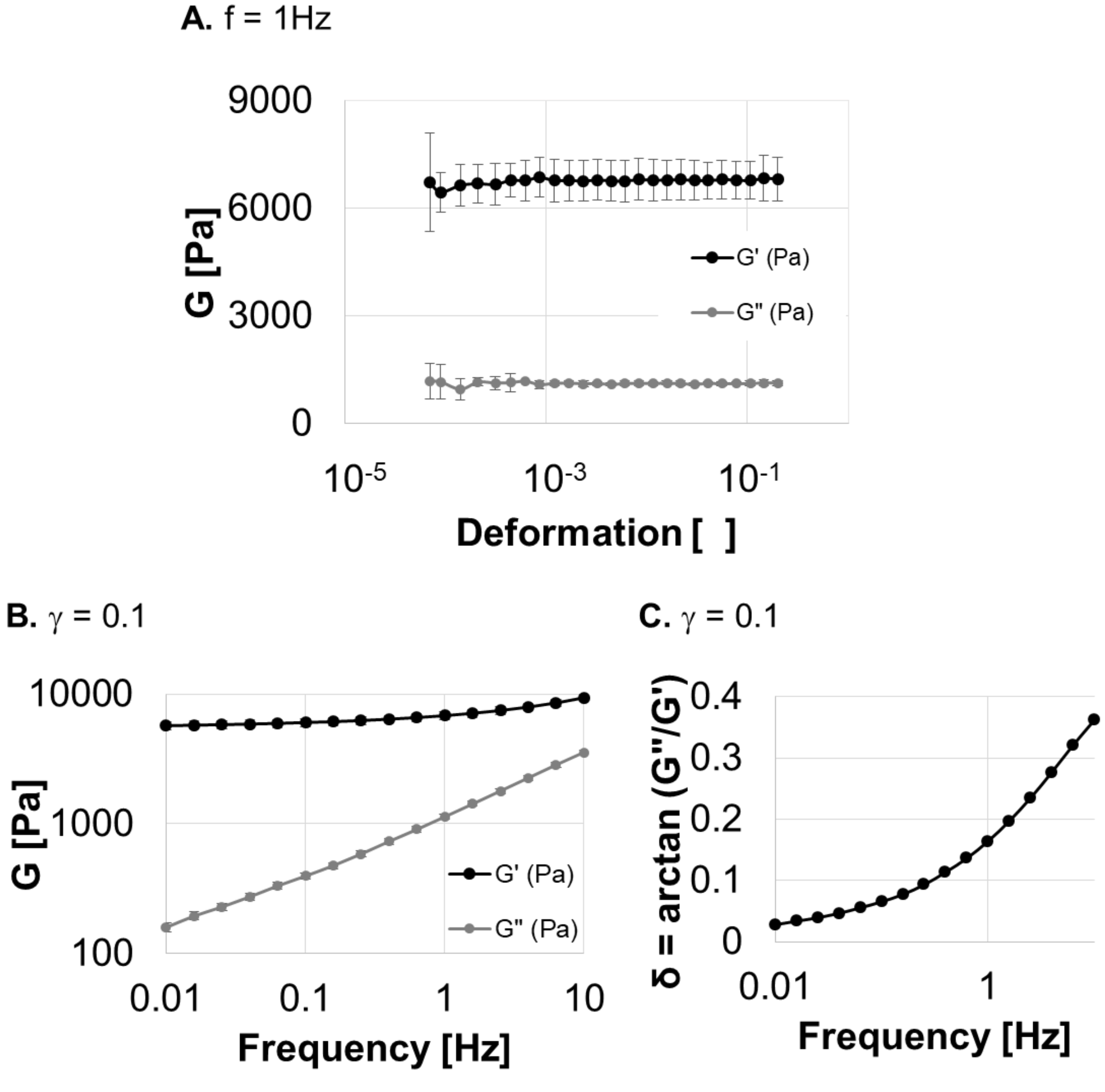

Fig. S1: Rheology of the soft PDMS. Shear storage modulus G' and loss modulus G" of the soft PDMS were measured with a rheometer used in parallel plane geometry on patches of 20mm diameter and 2mm thickness. (A) The amplitudes of γ were varied between 0.01% and 20% while the frequency f was fixed at 1Hz. (B) The shear deformation was then fixed at γ=10%

and the frequency varied between 0.01Hz and 10Hz. (C) The phase angle $\delta = Arctan\left(\frac{G"}{G'}\right)$ was calculated for the frequencies of interest so as to verify the domination of elasticity in the mechanical behaviour of the substrate (δ closer to 0 than $\pi/2$).

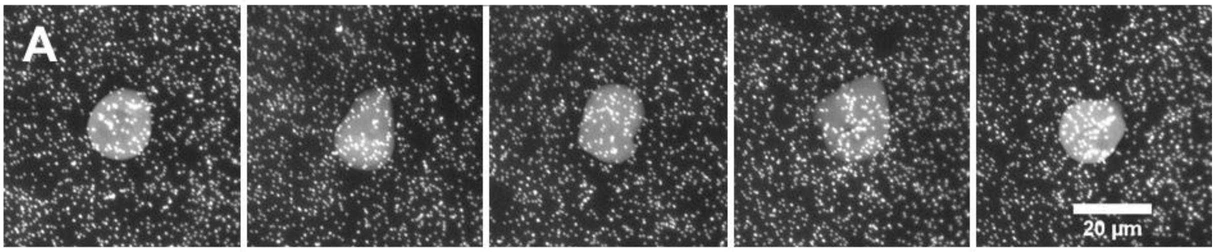

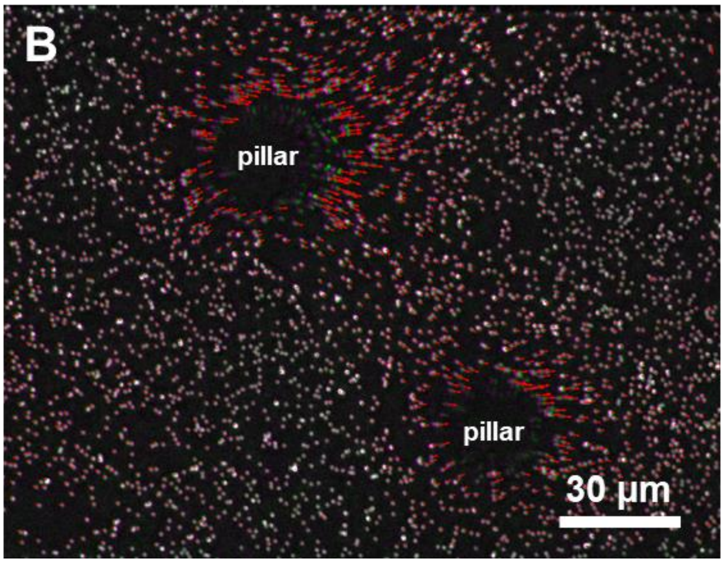

Fig.S2: The fluorescent beads embedded under the surface of the substrate have a homogeneous distribution and density, including above the pillars, as seen on samples imaged upside down (A). This allows particle tracking with high accuracy (20nm) and spatial resolution (<5µm) (B).

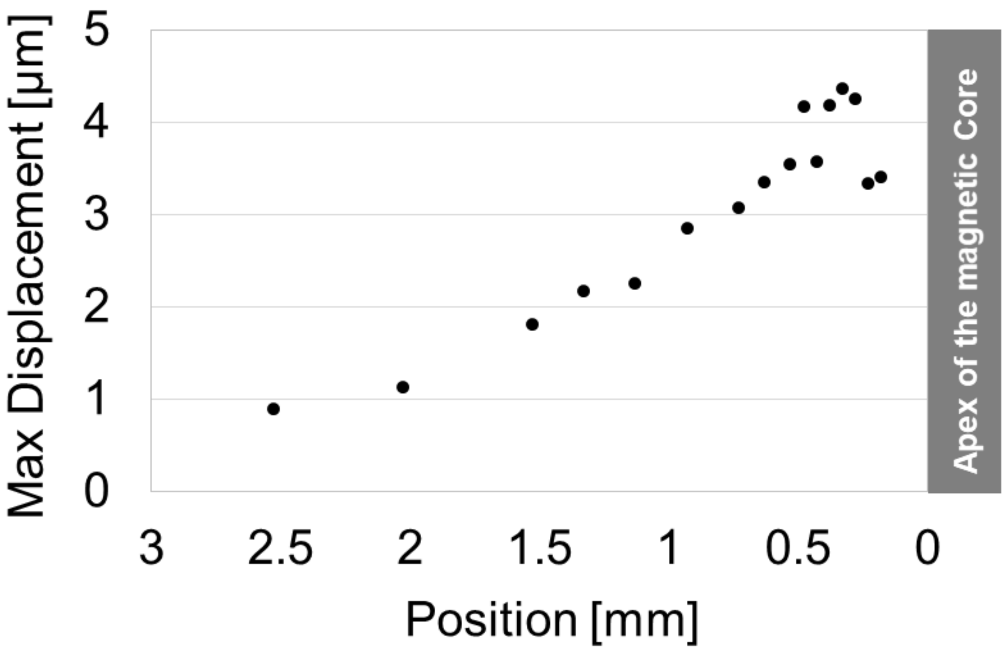

Fig.S3: Influence of the position of the pillar with respect to the electromagnet. The maximum displacement induced by the actuation of a pillar was measured as a function of the distance to the pole piece of the closest electromagnet.

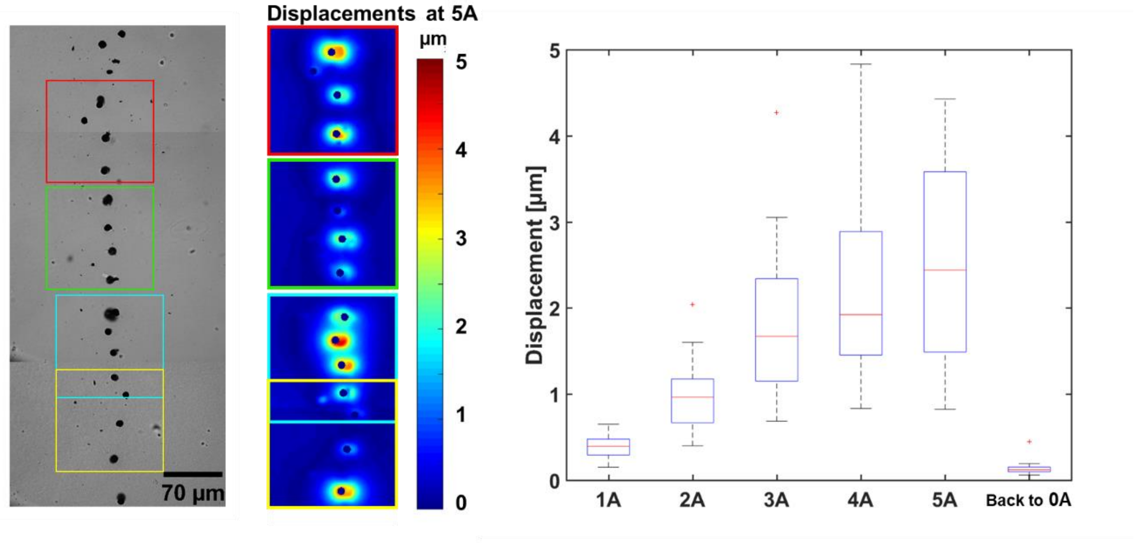

Fig.S4: Systematic analysis of a series of 15 pillars of the same row positioned 1.5mm away from the pole piece (Bz~20mT) and experiencing an incremental actuation (from 0 to 5A in the coils). Displacements were measured for all the pillars and the results plotted as a function of the current in the electromagnets.

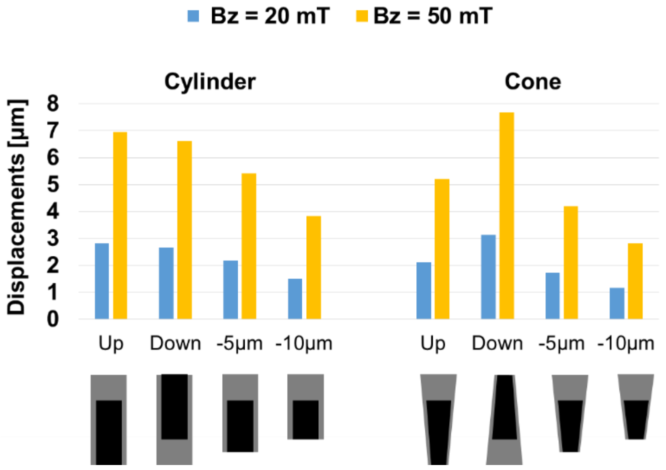

Displacements [µm]			
**Cylinder**	*Bz = 0 mT*	*Bz = 20 mT*	*Bz = 50 mT*
Up	0.3	2.8	6.9
Down	0.1	2.7	6.6
Up -5µm	0.4	2.2	5.4
Up -10µm	0.3	1.5	3.8
**Cone**	*Bz = 0 mT*	*Bz = 20 mT*	*Bz = 50 mT*
Up	0.5	2.1	5.2
Down	0.7	3.1	7.7
Up -5µm	0.4	1.7	4.2
Up -10µm	0.3	1.2	2.8

Fig.S5: Influence of the geometry of the pillar on the resulting actuation. 3D magneto-mechanical simulations were performed on core-shell pillars with the shape of a cylinder or a cone, and positioned with the head of iron towards the surface (up) or towards the coverslip (down).

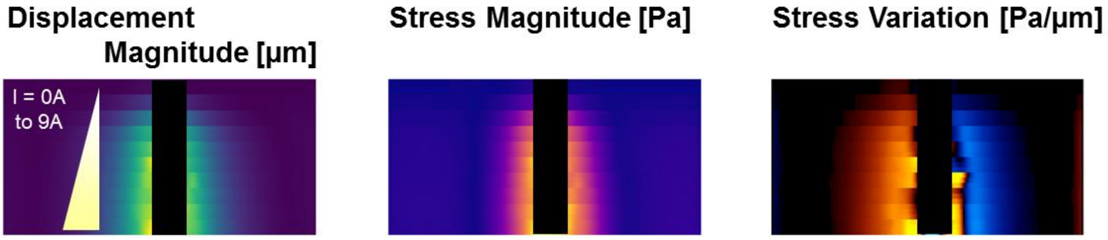

Fig.S6: Variation of current input. Profiles were extracted from deformation, stress and stress variation fields of the respective maps, shown in Figure 4, derived at different intensities between 0 and 9A, and displayed as kymographs.

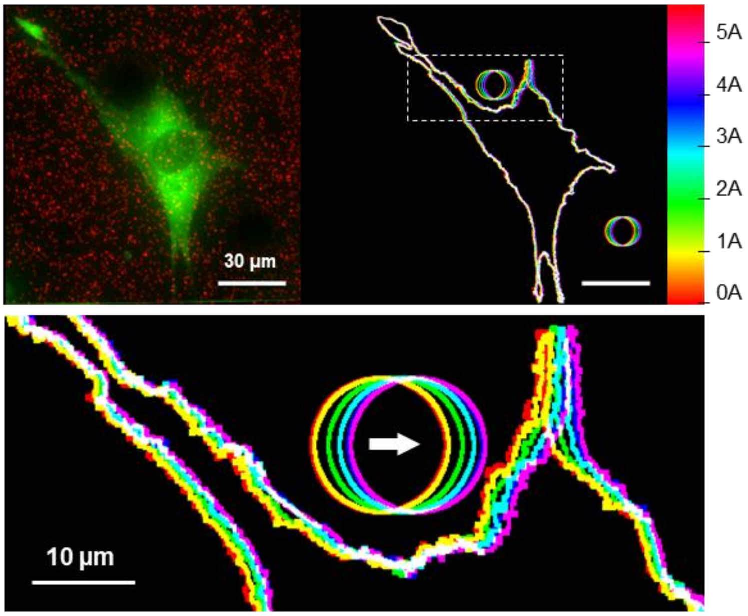

Fig.S7: Fluorescence images of a NIH3T3 vinculin eGFP fibroblast (green) and the beads spread under the surface (red) were used to draw the contours of the cell and the pillar respectively to highlight local deformations induced at different intensities of the field. The contours of the cell are mostly overlapping, except in the close vicinity of the pillar, where displacements from 5 to 0µm within about 30µm effectively deform the cell locally (zoom). The progressive local shift of the contours highlights the possibility to tune the degree of mechanical stimulation of the cell by adjusting the intensity of the current input in the electromagnets.

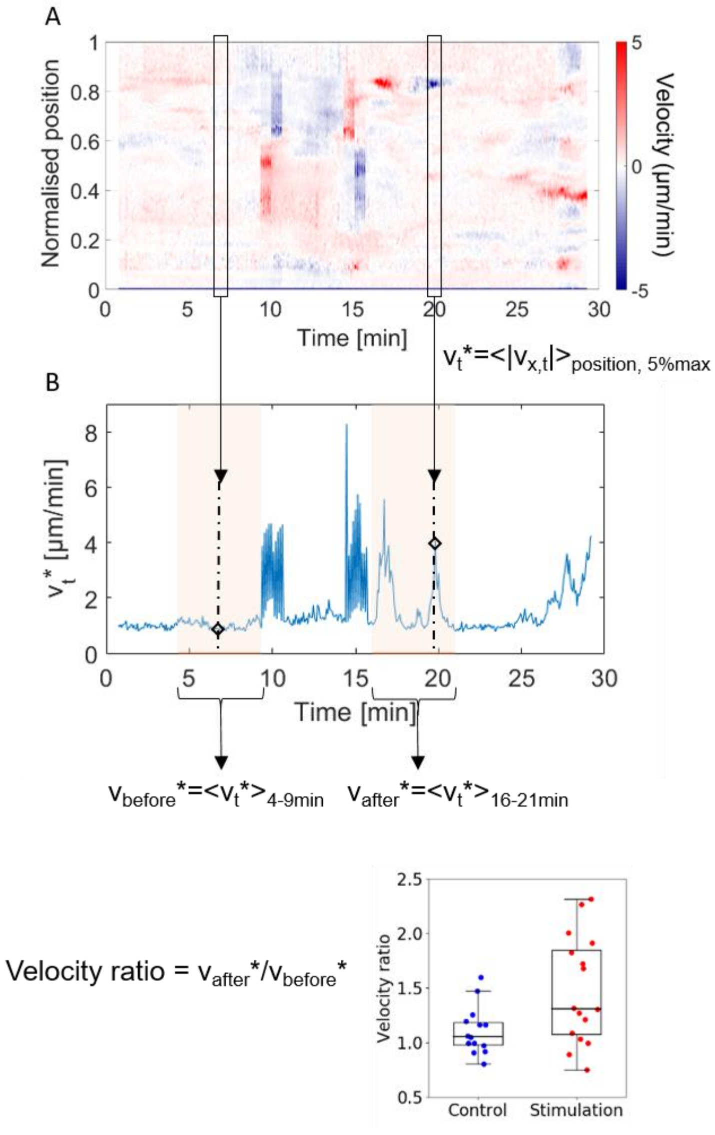

Fig.S8: Method to determine the ratios of protrusion velocity before and after stimulation. (A) Kymograph representing the normal velocity along the perimeter of the cell during the time of the experiment. A protrusion with a positive velocity (directed outwards) was plotted in red, a negative one (inwards) in blue. At the beginning and at the end of the stimulation period (10-15 minutes), large red and blue streaks appear in the area where the cell was deformed. This is also present before and after the stimulation due to the calculation of the velocity that impacts 10 frames after and before the considered frame. This velocity was spatially-averaged along the perimeter at a given time considering only the 5% maximal absolute values to obtain Fig. S8B. To obtain a single value per cell representing the possible change in protrusive activity after the stimulation, this velocity v_t* was then averaged over 5 minutes before and after the stimulation period, and the ratio was taken (1.7 for this cell). Hence, each point in the boxplot represents the response of a single cell.

Movie S9: Deformation of a fluorescent cell. A vinculin eGFP NIH3T3 fibroblast cell (green) was spread close to a pillar on a magneto-active substrate and actuated by electromagnets powered by a current of 0 to 5A. Fluorescent beads (red) highlight the deformation of the surface.

Movie S10: Cellular response of a vinculin eGFP NIH3T3 fibroblast to a dynamic stimulation, epifluorescence movie of the Vinculin-eGFP signal (left). During the first 10 minutes, the cell was at rest, then the magnetic pillar was actuated at 0.25Hz during 10 minutes and finally the cell was imaged for another 15 minutes. New protrusions are visible after the mechanical stimulation. In the middle part is shown an overlay of the TFM bead fluorescence after removal of the cell and with the pillar at rest (magenta) and of the beads during the acquisition (green). The pillar is located where the beads are invisible (top right), its movement is clearly visible during the stimulation. On the right side is shown the live TFM movie, for each frame the TFM map was calculated by taking as reference the bead image with no cell and pillar at rest. During the stimulation, large changes in stress are visible close to the pillar but also more subtle changes are visible on the opposite side of the cell.

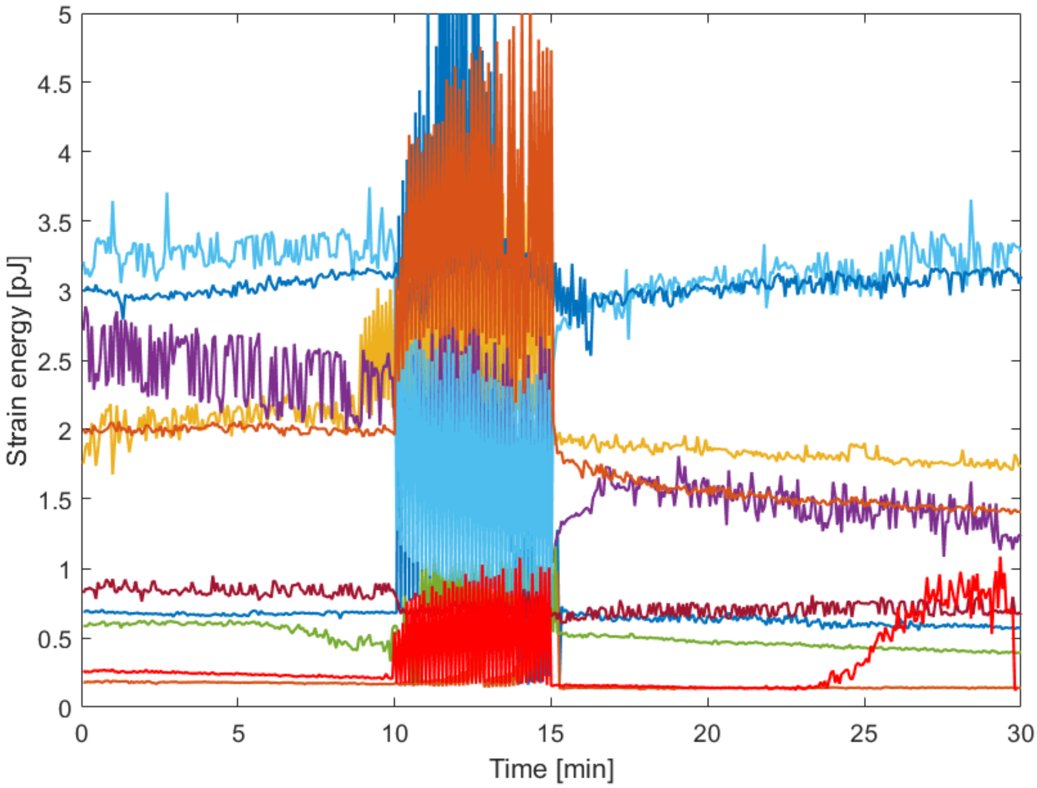

Figure S11: Strain energy of 10 different cells submitted to same protocol of mechanical stimulation consisting of 10 minutes rest, 5 minutes stimulation and 15 minutes rest. The stimulation period is clearly visible between times 10 and 15 minutes, but no clear trend on a cellular mechanical reaction to the dynamic deformation can be seen.
